# Peptide Mapping, In Silico and In Vivo Analysis of Allergenic Sorghum Profilin Peptides

**DOI:** 10.3390/medicina55050178

**Published:** 2019-05-21

**Authors:** Chandra Sekhar Bokka, Ganesh Kumar Veeramachaneni, V. B. S. C. Thunuguntla, Janakiram Bobbillapati, Jayakumar Singh Bondili

**Affiliations:** 1 Department of Biotechnology, Koneru Lakshmaiah Education Foundation, Green fields, Vaddeswaram, Guntur Dist., Andhra Pradesh 522 502, India; urschandu18@gmail.com (C.S.B.); ganesh.vgk55@gmail.com (G.K.V.); balasaichaitanya@gmail.com (V.B.S.C.T.); 2 Department of Microbiology, NRI Medical college and General Hospital, Guntur Dist., Andhra Pradesh 522 503, India; johnson.ratnam@gmail.com

**Keywords:** Sorghum, pollen allergen, profilin, peptide mapping, modeling, Th1 and Th2 cytokine

## Abstract

*Background and objectives*: Nearly 20–30% of the world’s population suffers from allergic rhinitis, among them 15% are progressing to asthma conditions. *Sorghum bicolor* profilin (Sorb PF), one of the panallergens, was identified, but the allergen specificity is not yet characterized. *Materials and Methods*: To map the antigenic determinants responsible for IgE binding, the present study is focused on in silico modeling, simulation of Sorb PF and docking of the Sorb PF peptides (PF1-6) against IgG and IgE, followed by in vivo evaluation of the peptides for its allergenicity in mice. *Results*: Peptide PF3 and PF4 displayed high docking G-scores (−9.05) against IgE only. The mice sensitized with PF3 peptide showed increased levels of IL5, IL12, TNF-alpha, and GMCSF when compared to other peptides and controls, signifying a strong, Th2-based response. Concurrently, the Th1 pathway was inhibited by low levels of cytokine IL2, IFN-γ, and IL-10 justifying the role of PF3 in allergenic IgE response. *Conclusions*: Based on the results of overlapping peptides PF3 and PF4, the N-terminal part of the PF3 peptide (TGQALVI) plays a crucial role in allergenic response of Sorghum profilin.

## 1. Introduction

Nearly 20–30% of the world’s population is suffering with allergic diseases viz., allergic rhinitis, atopic dermatitis, and bronchial asthma [1]. Due to drastic changes in climatic conditions and lifestyle, the prevalence of allergic diseases has significantly increased over the years. There are many different triggers of allergy and pollen grains of tree, grass, and weed plants are one of the major causative agents for respiratory allergic diseases, in particular asthma and rhinitis [2]. Allergenic pollen refers to the complex of several molecules, which includes major and minor allergens. More than 50% of the patients were prone to major allergens, while minor or panallergens sensitization was ~5–40% [3]. Profilin, an allergic protein of 12–15 kDa, was identified in tree, grass, weed pollens, latex, and plant-derived foods [4]. Due to its highly conserved sequence and three-dimensional structure, IgE specific to profilin may cross-react with homologs of almost all the known plants. Activated T helper-2 (Th2) lymphocytes produce Cytokines, IL (interleukins)-3, -4, -5, -9, -13 and GMCSF (granulocyte-macrophage colony-stimulating factor), which in turn activates eosinophils, mast cells, and IgE production. This mechanism resulted in the development of chronic allergic inflammatory disorders like reversible airway obstruction, airway inflammation, and hyper-responsiveness [5,6,7,8]. Although structurally several allergens have been characterized, common features predicting the potential of an allergenic protein are still unidentified [9]. Therefore, IgE epitope identification becomes crucial for better understanding the occurrence of allergenicity, and has direct impact on the development of therapeutic approaches for specific allergens. Sorghum plant allergenicity in India was reported to be ~54.9% [10]. In our previous work, the *Sorghum bicolor* profilin (Sorb PF) gene was identified based on homology, and an allergenicity score of 1.149 was reported based on in silico Algpred screening of known allergenic profilin sequences [11]. It has been well demonstrated that, B cell epitopes of allergens can well be mapped with synthetic peptides. Linear or conformational B cell epitopes situated on the surface of the allergen mostly accessible to immunoglobulin are well recognized by IgE antibodies [12].

The present work focused on the in silico molecular characterization of *Sorghum bicolor* profilin peptides. The in vivo approach was to screen and identify B cell epitopes by evaluating the IgE raised against each peptide in mice along with cytokines elicited either in the Th1 or Th2 pathway, responsible for allergenicity.

## 2. Materials and Methods

### 2.1. Homology Modeling and Evaluation

The Profilin sequence was retrieved from NCBI (Accession: KC427125, GenBank: AGN33439.1). Schrodinger software(San Diego, CA, USA) was used to develop the three-dimensional structure of the antigen using the Prime homology modeling tool. The application is bundled with blast tool for searching template, family search and secondary structure prediction tool, i.e., SSPro and PsiPred tools [13]. The built model was evaluated using the online tools ERRAT score [14] and PROCHECK [15].

### 2.2. Model Refinement

Based on the results obtained from the evaluation tools, the model was further refined by molecular dynamic simulations with the help of a Desmond module [16,17]. Simulations were executed in two steps: (1) the system builder created an environment for building the model by setting all the parameters to default. (2) Molecular dynamic simulations: the model with the environment created in the first step was imported, default relaxation protocol was applied, and the simulation run was set to 50 ns. The final model was analyzed using Root-mean-square deviation (RMSD) and Root-mean-square fluctuation (RMSF) parameters.

### 2.3. Ag–Ab Docking Studies

To know the binding site amino acids of the profilin antigen with IgE antibody, Ag–Ab docking studies were carried out. To implement this, a bioluminate module protein–protein docking application was used with the default parameters and with no constraints [18].

### 2.4. Peptide Docking Studies

The binding modes of the designed peptides against IgE antibody were analyzed using the Peptide docking application of the Bioluminate module of Schrodinger suite. The protocol involved various steps like protein preparation [19], receptor grid generation, peptide conformers generation, predocking, and docking.

### 2.5. Animal Maintenance

Six-week-old female BALB/c mice were bred and maintained in Gentox Animal House, Hyderabad. They were maintained in a conventional animal room with 12-h dark/light cycles and supplemented with pelleted pathogen-free food and water. Ethical Committee for Animal Experiments approved the experimental procedure (CPCSEA-1242/BC/08, dt.08/02/2013).

### 2.6. Peptide Immunization

A group of (*n* = 6) female BALB/c mice per peptide were sensitized by intraperitoneal (I.P) injections containing 100 µg of peptide adsorbed to the 100 μL of Alum (Imject Alum, Pierce Biotechnology, Rockland, ME, USA) on 0, 7 and 14th day. On 21st and 35th day, blood samples were withdrawn by retro-orbital bleeding [20]. The collected blood samples were kept for 30 min at room temperature to coagulate and then centrifuged for 10 min at 1000 g. After separation, serum was stored at −20 °C until used for analysis.

### 2.7. Overlapping Peptide Mapping and Synthesis

The 14-mer amino acid peptides were designed with an overlap of 7 amino acids using overlapping peptide fragment library software (Sigma Aldrich, St. Louis, MO, USA). The designed peptides were commercially synthesized on a Fmoc system (JPT Peptide Technologies GmbH, Berlin, Germany). All the peptides were supplied in the form of lyophilized powder with a purity of >95% analyzed by ultra HPLC and LC-MS/MALDI-MS (JPT Peptide Technologies GmbH, Berlin, Germany).

### 2.8. Cytokine Assays

Serum cytokine profiling was performed using a commercially available multiplexed kit: Bio-Plex Pro™ Mouse Cytokine Th1/Th2 Assay (Biorad Mouse Multi-Cytokine Detection System; Biorad Laboratories, Hyderabad, India). In brief, the serum was diluted in a 1:5 ratio and simultaneously measured for the levels of eight cytokines: IL-2, IL-4, IL-5, IL-10, IL-12 (p70), GMCSF, TNF-α (tumor necrosis factor-alpha), and INF-γ (interferon-gamma) as per the manufacturer’s protocol (Bio-Rad, Hyderabad, India). Cytokine concentrations were quantified using Bioplex protein array system (Bio-rad, Hyderabad, India) following the manufacturer’s instruction. The assay sensitivity was less than 10 pg/mL and ranged from 0.2 to 32,000 pg/mL with inter and intra assay cumulative value (CV) of less than 10% [21].

### 2.9. Total IgE and IgG Assays

The total IgE level was determined in the mouse serum using MILLIPLEX^®^ MAP Mouse IgE Single Plex Magnetic Bead Kit (Merck Millipore, Banglore, India) as per the manufacturer’s protocol and the samples were analyzed using the Luminex Magpix (Merck Millipore, Banglore, India) [22]. The total IgG level in the mouse serum was measured using the Amplex ELISA development kit (San Francisco, CA, USA) for mouse IgG as per the manufacturer’s protocol. All the samples were analyzed at 405 nm using ELISA plate reader. The results obtained were means of duplicate determination with variation less than 10%. Further, the IgE and IgG concentrations were determined by comparing the mean OD values of the tested sera with the mean OD values of the standard. IgE and IgG titers were calculated by multiplying the dilution factor of the test sera and expressed in ng/mL.

### 2.10. Statistical Analysis

To calculate statistical functions, the GraphPad Prism v.7.0 analysis program (San Diego, CA, USA) was used. The median of the parameter, standard deviation (SD) and the arithmetic mean were calculated. The nonparametric two-tailed *t*-test Two-Sample Assuming Unequal Variances was applied to compare measurable characteristics between groups. The differences were considered significant at *p* < 0.05.

## 3. Results

### 3.1. Homology Modeling, Evaluation, and Refinement

**Figure 1:**
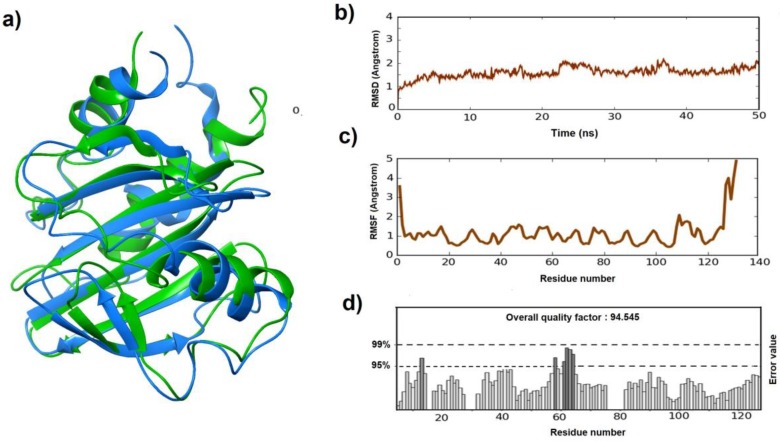
Homology model of *Sorghum* profilin and validation: The model was built using PDB: 5FDS as a template. (**a**) Built model before (green) and after (blue) simulations (**b**) Deviations graph of profilin after simulations (**c**) Fluctuations graph shown by the profilin residues during simulations (**d**) ERRAT score of the built model after simulation.

The allergen protein 3d structure of Sorghum profilin was determined using the prime homology modeling application of the Schrodinger suite. Protein Data Bank (PDB) structure 5FDS (crystal structure of the monomeric allergen profilin of Hevea brasiliensis): the top hit from the blast analysis was selected as a template and energy based model was developed (Figure 1a). The structural alignment of the model was evaluated through Ramachandran plot and ERRAT score. From the Ramachandran plot, it was observed that most of the amino acids (91.4%) were in most favored regions, 7.6% of the modeled profilin residues in additional allowed regions, and the remaining were found in generously allowed regions (Figure S1a). The ERRAT quality factor was 81.513, specifying that the model predicted was good but needs to be refined (Figure S1b). The model was further subjected to refinement with the help of molecular dynamic simulations to enhance the quality.

Desmond molecular dynamic simulation protocol was employed for a period of 50 ns, and the resulting trajectory file was analyzed to verify the deviations and fluctuation in the modeled protein. Deviations (RMSD) and fluctuations (RMSF) graphs reported that the overall deviations were in between 1.0 Å and 2.1 Å. Major inclinations and declinations in the deviations were observed from 10 ns and were continuous till to the end of simulation time ranging between 1.5 Å to 2.0 Å (Figure 1b). While the fluctuations are found between 3.7 and 5.0 Å, the highest fluctuations made by the residues were detected in the tail-end loop position (Figure 1c). In general, the tail-end residues fluctuate more to attain a stable conformational state and loop regions produced more fluctuations compared to other types. All the other residues fluctuated in between 0.5 to 1.5 Å. The majority of the residues were reported less than 1.0 Å, which indicates that the modeled protein was in a stable conformational state.

### 3.2. Antigen–antibody Binding Studies

To identify antigen–antibody interacting sites, an IgE-Profilin (protein–protein) docking study was performed. IgE antibody (PDB ID: 2VXQ) was retrieved and prepared by using the protein preparation wizard. Concurrently, all the simulated trajectory frames of the modeled antigenic protein, profilin were clustered based on the energy and deviations. The cluster center frame showing minimal energy, deviations and fluctuations was chosen for docking studies. Tail-end sequences of the antigen were found intact with the paratope region of the antibody by the end of docking studies. To validate the importance of other amino acids in the antigen, the sequence was divided into overlapping peptides.

### 3.3. Peptide Docking Studies

**Figure 2:**
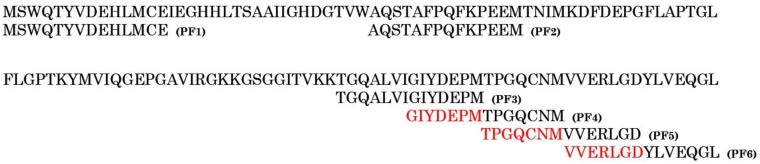
*Sorghum* profilin antigen sequence (GenBank: AGN33439.1) aligned with identified peptides (PF1-6). Peptides (PF3-6) have seven overlapping amino acids in each peptide at the C-terminal.

**Figure 3:**
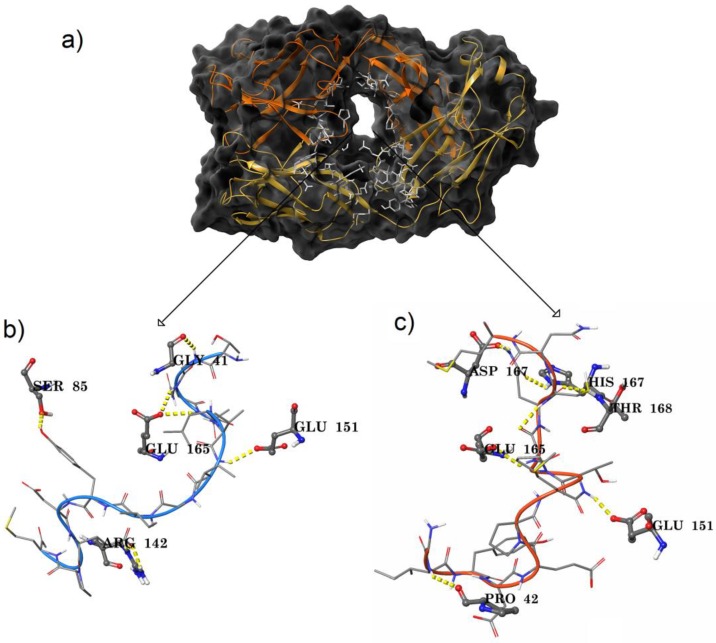
Paratope interactions: (**a**) Paratope region of IgE; heavy chain in orange color and light chain in yellow color. (**b**) PF-3 peptide binding interaction profile with IgE. (**c**) PF-4 peptide interaction with the IgE paratope region residues.

The profilin sequence was processed using overlapping peptide fragment library software and six different 14-mer peptides were designed (PF1-6) with an overlap of seven amino acids (Figure 2). Initially, the amino acids covering the paratope region of the IgE antibody were identified using the Sitemap module (Figure 3a): 21 residues from the heavy chain and 24 residues from the light chain were reported (Table 1). The interaction of the six peptides (PF1-6) with both IgE and IgG antibodies was studied to check their specificity with the help of peptide docking application. Fab part of IgG was retrieved from protein data bank (4J4P) following the same docking protocol. The top ranked pose of each peptide with reference to antibody were shown (Table 2). Peptide PF1 produced interactions with the IgG antibody alone and failed to produce interactions with IgE. The PF2 peptide had a very low G-score compared to the other peptides against IgE and, with IgG, the G-score was similar to PF6. PF 1 and 2 showed no or less interaction with IgE and, concurrently, PF 5 and 6 had a similar type of activity against both the antibodies based on the G-scores. It was evident through docking studies that PF3 and PF4 peptides made their impact differently against the two antibodies exhibiting low G-scores with IgG when compared to IgE.

The PF3–IgE complex showed five hydrogen bonds. Out of which, four were maintained with the light chain and the remaining H bond was with the heavy chain. Starting residues, G of PF3 linked with Gly 41, and Q and A residues in the peptide sequence produced individual bonds with Glu 165. Tail-end peptide residues: Y produced a hydrogen bond with Ser 85, and E formed two hydrogen bonds with Arg 142 of the light chain of the IgE. The only hydrogen bond observed between peptide and heavy chain was shared between I residue in the starting position of PF3 with Glu 151 (Figure 3b). Peptide PF4 was found to have six hydrogen bonds with IgE. Heavy chain amino acids: Pro 42 formed a hydrogen bond with the I residue of the peptide, Glu 151 with the T residue of PF4, and Asp 167 and Thr 168 with Q residue of the peptide. While light chain amino acids Glu 165 bonded with G residue positioned at the N-terminal of the peptide sequence and His 167 with Q residue of PF4. Out of six hydrogen bonds, the Q residue of the peptide showed a maximum of three interactions (Figure 3c). Based on the in silico analysis, profilin PF3 and PF4 peptides were identified to have specific IgE paratope interactions and their binding poses were represented in Figure S2. All the peptides (PF1-6) were further validated by in vivo studies in BALB/c mice.

### 3.4. Cytokines

**Figure 4:**
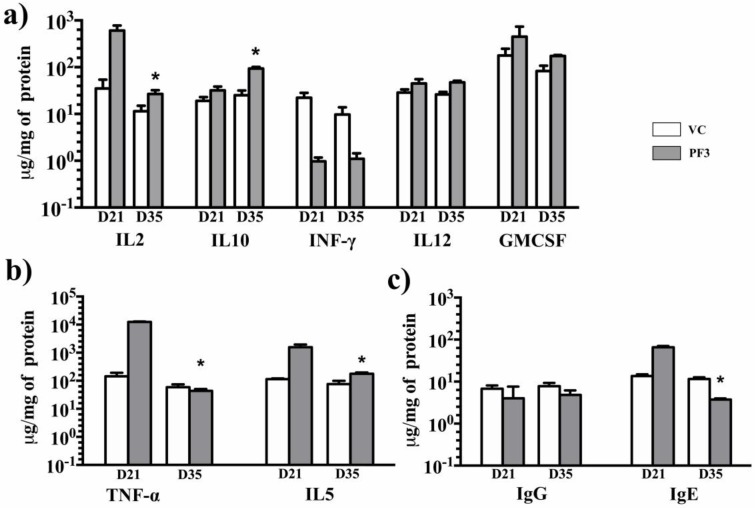
PF3 Cytokine data: PF-3 peptide sensitized mouse serum samples were estimated for (**a**) IL2, IL10, INF-γ, IL12, and GMCSF data on the 21st and 35th days. (**b**) TNFα and IL 5 concentration decreased on the 35th day. (**c**) IgG and IgE profiles of PF3 sensitized mice. The data shown is mean ± SD. *represents significance at 95% confidence. VC: Vehicle Control. D21: Day21, D35: Day35.

To clarify the mechanism involved in eliciting the allergenic reaction by Sorghum profilin, we examined the cytokine levels in serum of mice that were stimulated with PF1, PF2, PF3, PF4, PF5, and PF6 peptides in vivo. The mice sensitized with PF3 peptide showed significantly increased levels of IL5, IL12, TNF-α, and GMCSF when compared to non-sensitized mice and vehicle control (Figure 4), signifying a strong Th2-based response. The total cytokine concentration of IL5, IL12, TNF-α, and GMCSF were found to be increased (Table 3) on 21st day compared to vehicle control. However, on the 35th day, IL5, TNF-α, and GMCSF showed significant decrease while IL12 concentration was maintained almost at the same level. Concurrently, the Th1 pathway cytokine IL2 level was though found elevated significantly on the 21st day, gradually decreased by 35th day when compared to vehicle control. The IFN-γ concentration was sustained at a very low level on the 21st day and showed no significant variation until 35th day. Overall, the Th1 pathway cytokines (IL-2 and IFN-γ) remained at low levels until the 35th day, thereby promoting the production of IgE (Figure 4). The IL-10 concentration was also found to be raised, but limited, when compared to vehicle control and other cytokines. There was no significant difference found in mice sera with other peptides (PF 1, 2, 4, 5, and 6) injected when compared to controls.

### 3.5. Total IgE and IgG

We detected higher serum total IgE levels in the PF3 (65.63 µg/mg of protein) and PF4 (18.20 µg/mg of protein) peptides sensitized mice on the 21st day when compared to the vehicle control (13.57 µg/mg of protein) and other peptides (PF1, PF2, PF5, and PF6). The levels were significantly decreased for both peptide PF3 (*p* = 0.002) and PF4 (*p* = 0.032), 3.74 and 3.6 µg/mg of protein, respectively, on the 35th day. Concomitantly, the total IgG concentration remained almost at the same levels and was found to be 4.01 and 4.45 µg/mg of protein on the 21st day and 4.12 and 4.46 µg/mg of protein on the 35th day respectively. 

## 4. Discussion

Due to lack of crystal structure, the allergen homology 3D-model was built for identifying the accessible epitopic regions on the surface of the Sorb PF allergen. In addition, to understand the interaction profiles between the allergen and IgG and IgE antibodies, the antigen sequence was divided into overlapping peptides and their binding modes against both the antibodies was studied. RMSD and RMSF from the simulation results were found to be in the acceptable range of 1 to 3 Å. The refined model ERRAT score increment (Figure 1d) justifies the increase in overall modeled protein stability [14]. The enhancement in the model score was mainly because of the refinement of loop regions present in the model. The difference in the RMSD scores before and after simulation model was found to be 3.5 Å. In general, for small globular proteins, changes in the order of 1 to 3 Å are acceptable. However, changes much larger than that indicate that the protein is undergoing a large conformational change during the simulation. 

From the Ag–Ab docking results, it was observed that tail-end positions were more focused towards the antibody in forming a complex while the central domains of the fully folded secondary structure of the modeled protein failed to produce interactions in any of the orientations. This prompted for the design of the peptides and the antigen protein sequence was split into overlapping peptides [23] and their binding mode against both IgG and IgE antibodies was studied. Overlapping peptides docking studies showed that PF3 and PF4 peptides interacted differently against IgG and IgE. In line with previous studies, PF3 and PF4 peptides exhibited low G-scores with IgG when compared to IgE [24].

Type-I allergy pathogenesis is initiated by antigen-presenting cells (APC) and phagocytosis of allergens, thereby presenting antigen to naive T cells. Conventionally, of the four distinct populations of CD4+ T cells—T helper type 1 (Th1), T helper type 2 (Th2), T helper 17 (Th17), and regulatory T (Treg) cells [25]—the Th2 cells mainly produced interleukin IL-4 and IL-5, which promote IgE synthesis. Contrasting this, the Th1 cells mostly release cytokines, such as IL-2 and interferon (IFN-γ), and thereby prevent IgE production [26]. The equilibrium between Th1 and Th2 is considered to be important, for the development of allergic diseases and in immune homeostasis [27]. Regulatory T cells regulate the Th1–Th2 balance and suppress the allergic response [28]. Cytokine assay reported that the mice sensitized with PF3 peptide maintained IL12 steady state concentration by the end of 35th day, justifying the costimulatory role in the activation of Th1 cells while suppressing Th2 generation. Unlikely, the IL2 levels were not maintained high promoting the Th1 pathway. Concurrently, the role of IL10 is known to be potently immune-suppressive, which is vital for the development of peripheral tolerance to allergens and also protecting from exaggerated inflammatory responses in the host. The IL-10 concentration was found to be raised on the 35th day with regard to PF3, and there was no noteworthy difference found in mice with the remaining peptides. In the present study, an increased level of IgE was seen with the PF3 peptide when compared to IgG production. Th1 cells can induce cell-dependent immunity by producing IFN-γ, and also help in promoting immunoglobulin class switching mechanisms specific to IgG1 and IgG2A. Therefore, it is not surprising that the in vivo serum levels of IgG did not show any increase when compared to the control mice as quite low IFN-γ levels were detected in the mice.

Based on the above, only PF3 peptide was found responsible for the allergenic response following Th2 pathway. Though docking studies revealed two peptides PF3 and PF4 to be more significant with regard to binding modes against IgE and IgG, only the PF3 peptide showed increased Th2-based cytokine response when injected in mice (PF4 data: Figure S3); it also exhibited higher titers of IgE in mice when compared to PF4.

## 5. Conclusions

In conclusion, this study highlights the 3D modeling of Sorghum profilin and based on in silico and in vivo data of peptides screened, the N-terminal part of the PF3 peptide (TGQALVI) is identified to play a vital role in eliciting Th2 allergenic response and thereby leading to the production of IgE. The structural and cytokine data confirm that the PF3 peptide is responsible for allergenic response and could be helpful in developing the new diagnostic kits targeted towards allergen screening.

## Figures and Tables

**Table 1:** List of amino acids (residue number) identified in the paratope region of IgE antibody involved in hydrogen bond formation with PF3 and PF4 peptides.

Amino Acid Range	Heavy Chain	Light Chain
**0–50**	40, 42 (PF4), 44, 45	12, 38, 40, 41 (PF3)
**51–100**	91, 92, 93, 95	83, 84, 85 (PF3), 87, 100
**101–150**	112	101, 103, 104, 105, 140, 141, 142 (PF3), 143
**151–200**	151 (PF3 & PF4), 152, 153, 154, 155 163, 164, 165, 167 (PF4), 168 (PF4), 170, 171	163, 164, 165 (PF3 & PF4), 166, 167 (PF4), 168 (PF4), 173

**Table 2:** Peptides docking G-score against IgG and IgE. Highlighted (bold) amino acids of PF3 and PF4 showed hydrogen bond interactions with the paratope region of IgE.

Peptide	IgG	IgE
MSWQTYVDEHLMCE (PF 1)	−11.62	No binding
AQSTAFPQFKPEEM (PF 2)	−9.61	−3.21
T***GQA***LVIGI***Y***D**E**PM (PF 3)	**−5.54**	**−9.05**
***GI***YDEPM***T***PG***Q***CNM (PF 4)	**−5.37**	**−9.05**
TPGQCNMVVERLGD (PF 5)	−12.60	−12.05
VVERLGDYLVEQGL (PF 6)	−9.25	−10.63

**Table 3:** Th1 and Th2 pathway cytokine quantification data.

Serum	VC		PF3		*p*-Value
	D21	D35	D21	D35	(<0.05)
**IL2**	35.20 ± 19.02	11.42 ± 3.62	608.46 ± 172.30 ^#^	26.76 ± 5.36 ^#,^*	0.02
**IL12**	28.68 ± 4.91	26.07 ± 3.61	44.99 ± 10.20	47.39 ± 3.90 ^#^	-
**TNF-α**	143.33 ± 50.11	59.05 ± 15.54	12439.40 ± 445.69 ^#^	43.62 ± 6.87 *	0.0004
**INF-γ**	22.24 ± 5.96	9.73 ± 4.20	0.97 ± 0.20	1.10 ± 0.34 ^#^	-
**IL5**	114.87 ± 6.23	75.88 ± 6.23	1569.43 ± 375.16 ^#^	177.56 ± 19.73 ^#,^*	0.02
**IL10**	18.99 ± 4.01	25.13 ± 6.59	31.96 ± 6.56	93.59 ± 7.52 ^#,^*	0.004
**GMCSF**	177.79 ± 70.58	82.72 ± 24.72	449.23 ± 286.95	173.52 ± 8.27 ^#^	-
**IgG**	6.77 ± 1.32	7.78 ± 1.52	4.01 ± 2.58	4.12 ± 1.35	-
**IgE**	13.57 ± 1.36	11.53 ± 1.15	65.63 ± 4.65 ^#^	3.74 ± 0.24 ^#,^*	0.002

All data is represented in mean ± SD (*n* = 3); VC: Vehicle Control; D21: Serum cytokine levels on 21st Day; D35: Serum cytokine levels on 35th day; *p*-values represented for D21 vs. D35 data sets of PF3 induced groups. Significance determined by using *t*-test: Two-Sample Assuming Unequal Variances (two-tailed). *: significance between D21 and D35 of PF3 group; ^#^: significance between VC and PF3 groups on respective days.

## Supplementary Materials

The following are available online at https://www.mdpi.com/1010-660X/55/5/178/s1, Figure S1: (**a**) Ramachandran plot of Profilin homology model showing that 91.4% of amino acids are in the favored region and the rest in allowed regions. (**b**) ERRAT profile of the built profilin model before simulations. Figure S2: (**a**) Binding pose of PF3 sequence (**b**) Binding pose of PF4 sequence. Figure S3: Quantification of Cytokines: PF-4 peptide sensitized mouse serum samples were estimated for (**a**) IL2, IL10, TNFα, INF-γ, and IL5 (**b**) IL12, GMCSF, IgG, and IgE profiles of PF4 sensitized mice. The data shown is Mean ± SD (*n* =3 ). * represents significance at 95% confidence.
