# Supplementary material for: Peptide Mapping, In Silico and In Vivo Analysis of Allergenic Sorghum Profilin Peptides
Source: Medicina (Kaunas). 2019 May 21;55(5):178. doi: 10.3390/medicina55050178 (PMC6571906; doi:10.3390/medicina55050178)
Supplement: Supplementary file 1 [file medicina-55-00178-s001.pdf]

Supplementary Materials:

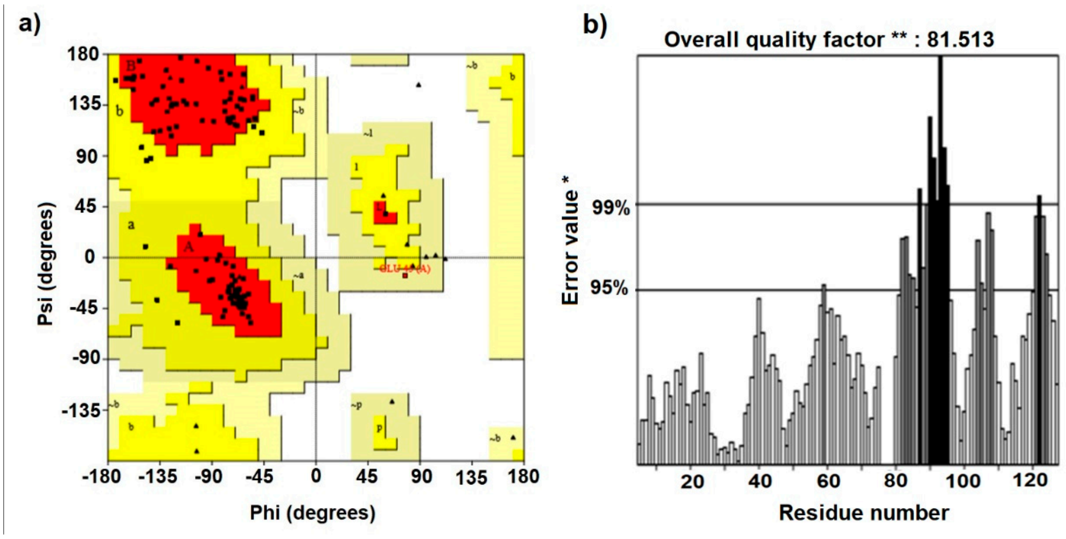

Figure S1: (**a**) Ramachandran plot of Profilin homology model showing 91.4% of amino acids are in favored region and rest in allowed regions (**b**) ERRAT profile of the built profilin model before simulations.

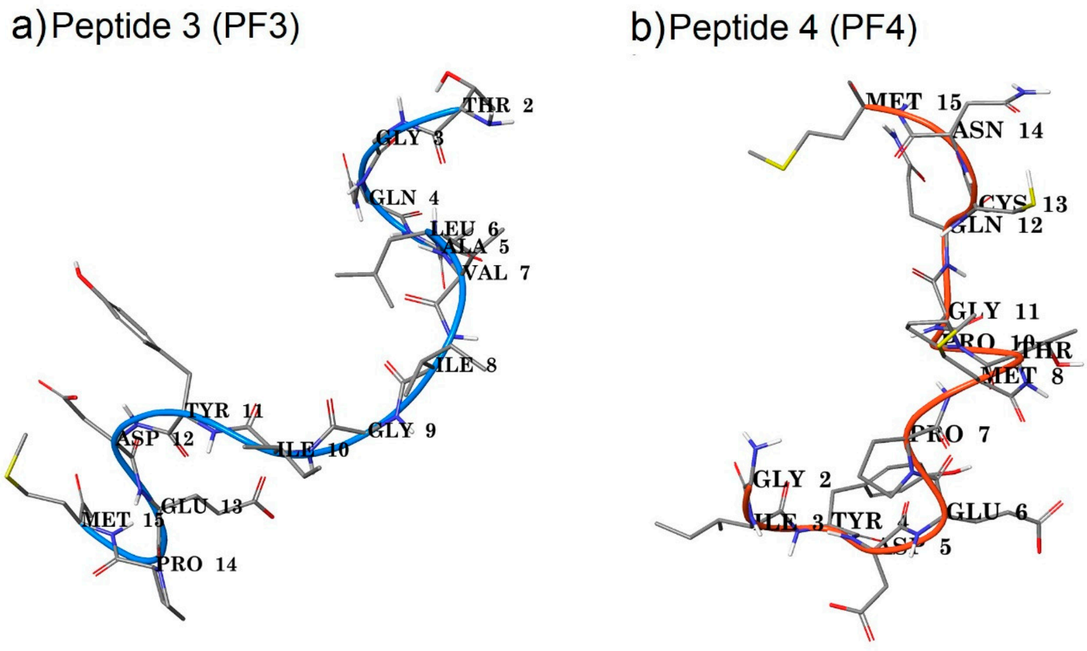

Figure S2: (**a**) Binding pose of PF3 sequence (**b**) Binding pose of PF4 sequence

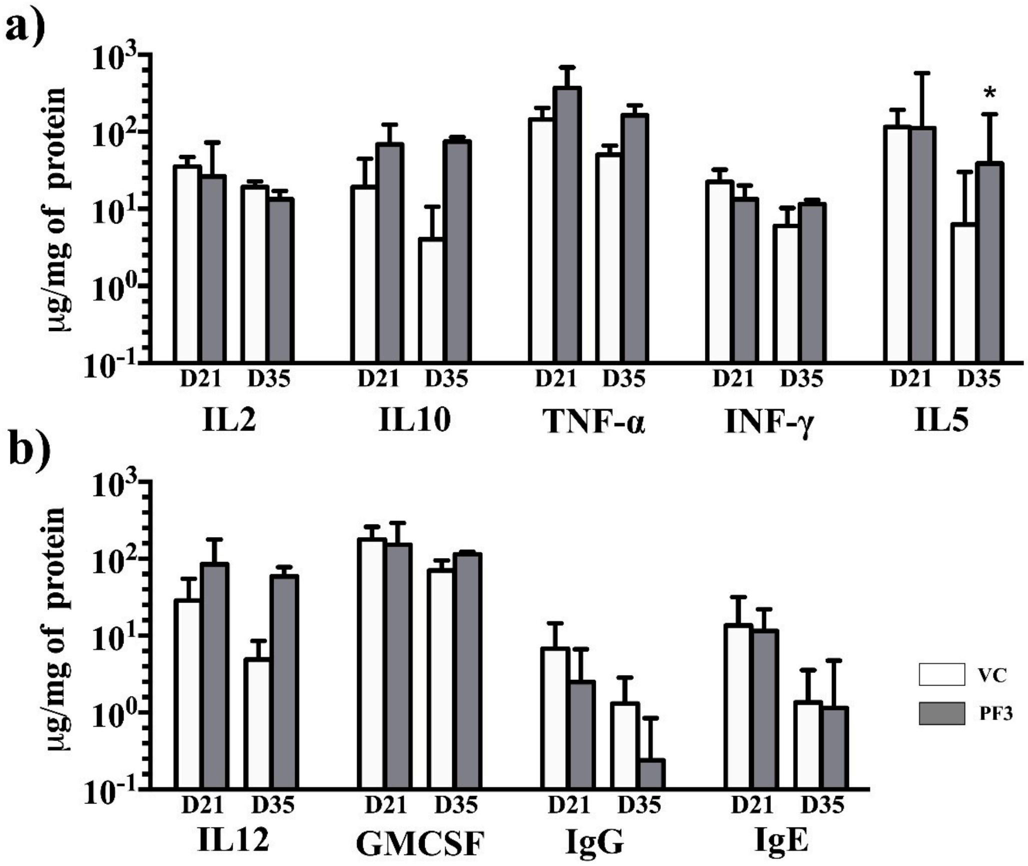

Figure S3: Quantification of Cytokines: PF-4 peptide sensitized mouse serum samples were estimated for (**a**) IL2, IL10, TNFα, INF-γ and IL5 (**b**) IL12, GMCSF, IgG and IgE profiles of PF4 sensitized mice. The data shown is Mean ± SD (n = 3). *represents significance at 95% confidence.
